# Editorial: The cellular stress response and physiological adaptations of corals subjected to environmental stressors and pollutants, volume II

**DOI:** 10.3389/fphys.2024.1473792

**Published:** 2024-08-19

**Authors:** Davide Seveso, Yohan D. Louis, Ranjeet Bhagooli, Craig A. Downs, Walter Dellisanti

**Affiliations:** ^1^ Department of Earth and Environmental Science, University of Milano Bicocca, Milano, Italy; ^2^ MaRHE Center (Marine Reseacrh and High Education Center), Magoodhoo, Faafu, Maldives; ^3^ Department of Biosciences and Ocean Studies, Faculty of Science and Pole of Research Excellence in Sustainable Marine Biodiversity, University of Mauritius, Réduit, Mauritius; ^4^ The Biodiversity and Environment Institute, Réduit, Mauritius; ^5^ Institute of Oceanography and Environment (INOS), University Malaysia Terengganu, Kuala Terengganu, Terengganu, Malaysia; ^6^ Department of Marine Science, Faculty of Fisheries and Marine Science, Diponegoro University, Semarang, Indonesia; ^7^ The Society of Biology (Mauritius), Réduit, Mauritius; ^8^ Haereticus Environmental Laboratory, Clifford, VA, United States; ^9^ Marine Biology Section, Department of Biology, University of Copenhagen, Helsingør, Denmark

**Keywords:** cellular stress, corals, biomarkers, climate change, anthropogenic impact

There is substantial evidence that coral reefs are suffering worldwide due to global climate change, anthropogenic pressures, and local stressors, which led to their rapid decline over the last few decades with bleaching causing most of this loss (Hughes et al., 2017; Hughes et al., 2018). In order to more accurately predict the impacts of global changes and develop conservation and stress mitigation strategies, efforts have recently increased in elucidating the cellular and molecular mechanisms underlying coral bleaching and other coral responses to environmental stressors (Helgoe et al., 2024). As sessile and long-lived animals that experience variable conditions, corals rely mainly on their cellular stress responses for acclimatization and adaptation (Drury, 2020). Moreover, since changes at the cellular level are the first detectable responses to environmental perturbations, the analysis of cellular biomarkers represents a useful diagnostic tool reflecting variations in cellular integrity and pathways before larger-scale processes are affected (Downs, 2005; Louis et al., 2020; Montalbetti et al., 2021).

Although recent and substantial advances in omics technologies have made the study of coral molecular processes more efficient, rapid, and accessible (Weis, 2019; Cziesielski et al., 2020), our understanding of coral cell biology remains inadequate (Oakley and Davy, 2018; Weis, 2019). This Research Topic aimed to expand this knowledge and bridge existing gaps. The articles presented here demonstrate how various physiological and molecular approaches and techniques can be adopted to understand the responses of coral holobionts to a multitude of stressors.

Sea surface temperature increase and heat waves are the primary drivers of coral bleaching and reef degradation worldwide (Hughes et al., 2018; Eakin et al., 2019). Therefore, mesophotic habitats often represent potential refugia for corals (Bongaerts et al., 2010; Muir et al., 2017). In their study, Tavakoli-Kolour et al. analyzed the photosynthetic efficiency (maximum quantum yield at photosystem II) and the bleaching conditions, via symbiotic microalgal density and chlorophyll concentrations, of mesophotic and shallow coral species subjected to different temperature scenarios reproducing different Degree Heating Weeks (DHWs). Their results indicated that mesophotic corals have a threshold temperature slightly lower or equal to that of shallow corals, suggesting that, although they can survive thermal stress below 4 DHWs, mass bleaching can occur above this threshold. Coral reefs at relatively high latitudes could also be potential refuges for corals (Camp et al., 2018; Dellisanti et al., 2023). However, corals living in such habitat could suffer from low-temperature stress, inducing bleaching (Tracey et al., 2003; Marangoni et al., 2021). Wei et al. explore the response of *Porites lutea* from a high-latitude coral reef in the South China Sea under acute (1–2 weeks) and chronic (6–12 weeks) low-temperature stress, by analyzing maximum quantum yields and transcriptomic profiles. Low temperatures inhibited photosynthetic efficiency and reduced energy production and calcification by down-regulating sugar metabolism and calcification-related genes. However, this was particularly observed during a short acute treatment, suggesting a possible coral acclimation to chronic low temperature.

Although thermal stress is recognized as the main cause of coral bleaching, high solar irradiance can also play a central role in this process by exacerbating the production of reactive oxygen species (ROS) (Roth, 2014; Courtial et al., 2018). Shading-based management interventions could therefore reduce coral bleaching risk. Butcherine et al. examined the effectiveness of intermittent shade on two coral species held at either optimum or high temperatures. The analysis of coral health condition through the bleaching assessment (chlorophyll *a*, and symbiont density), the photochemistry, and the use of antioxidant enzymes (SOD and CAT) as cellular stress biomarkers, suggested that intermittently shading corals for 4 h can mitigate the impact of thermal stress.

However, even extremely low light levels, mainly related to high sedimentation rate and turbidity, can induce coral bleaching and negatively impact coral metabolism (DeSalvo et al., 2012; Bollati et al., 2021; Tuttle and Donahue, 2022). Using transcriptomics, Lock et al. identified gene expression patterns and molecular pathways that may allow the massive coral *Porites lobata* to tolerate and persist to chronic and severe sedimentation in the turbid Fouha Bay (Guam), providing important insights into coral metabolic plasticity and acclimation to this stressor. In particular, alternative energy generation pathways may help to counteract low light and oxygen levels, the upregulation of apoptosis genes may maintain colony integrity, and increased expression of cellular communication genes may help corals respond to sediment-associated pathogens.

Molecular biomarkers are also used as a proxy for water quality and anthropogenic pollution. Tisthammer et al. employed enzyme-linked immunosorbent assays to evaluate stress responses in *P. lobata* along an environmental gradient in Maunalua Bay (Hawaii), revealing distinct protein expression patterns, especially those of ubiquitin and Hsp70, which correlate with anthropogenic stressor levels across the bay. Nardi et al. analyzed the ecotoxicological response of the Mediterranean coral *Madracis pharensis* to polycyclic aromatic hydrocarbons (PAHs) bioaccumulated from chronic oil leakage from a shipwreck in Cyprus. The high ROS scavenging capacity and the low functionality of detoxification processes associated with the glutathione-S-transferase enzyme suggested that *M. pharensis* has the capability to develop cellular and physiological adaptations to chemical-mediated stress. Morgan et al. focused on the synergistic, antagonistic, or additive effect of oxybenzone BP-3, the active ingredient in sunscreen, and ocean acidification (OA), on the expression profiles of 22 genes of interest (GOIs) in sea the anemone *Exaiptasia diaphana*. The collective antagonistic responses of GOIs associated with collagen synthesis suggested their role as candidate biomarkers of stress, while GOIs with synergistic and additive responses, such as serotransferrin-like (TF) and monocarboxylate transporters (MCTs) genes, respectively, were also identified.

Finally, cellular stress mechanisms are also known to be involved in coral response to biotic interactions (Seveso et al., 2012; Seveso et al., 2017). For example, using gel-filtration chromatography and liquid chromatography-tandem mass spectrometry, Suzuki et al. identified and characterized red fluorescent proteins (RFPs) and chromoproteins (CPs) in inflammatory pink lesions of *Porites* colonies subjected to the pink pigmentation response (PPR). The results suggested a possible differential role of these proteins in coral immunity despite their coexistence. Additionally, CPs, which are specifically expressed in PPR lesions, may serve as an antioxidant protection, providing new insights into the role of CPs in the coral immune response.

Considering that understanding how corals can genetically or physiologically adapt to environmental changes has become a global research priority, we believe that this Research Topic provides a more comprehensive view of the cellular mechanisms involved. It may encourage future advancements in this field and support strategies and tools to potentially reduce or mitigate the impacts of cellular stress in corals.
